# Up, Down, and All Around: Scale-Dependent Spatial Variation in Rocky-Shore Communities of Fildes Peninsula, King George Island, Antarctica

**DOI:** 10.1371/journal.pone.0100714

**Published:** 2014-06-23

**Authors:** Nelson Valdivia, María J. Díaz, Jorge Holtheuer, Ignacio Garrido, Pirjo Huovinen, Iván Gómez

**Affiliations:** 1 Instituto de Ciencias Marinas y Limnológicas, Facultad de Ciencias, Universidad Austral de Chile, Valdivia, Chile; 2 Programa de Doctorado en Biología Marina, Facultad de Ciencias, Universidad Austral de Chile, Valdivia, Chile; Institute of Ecology, Germany

## Abstract

Understanding the variation of biodiversity along environmental gradients and multiple spatial scales is relevant for theoretical and management purposes. Hereby, we analysed the spatial variability in diversity and structure of intertidal and subtidal macrobenthic Antarctic communities along vertical environmental stress gradients and across multiple horizontal spatial scales. Since biotic interactions and local topographic features are likely major factors for coastal assemblages, we tested the hypothesis that fine-scale processes influence the effects of the vertical environmental stress gradients on the macrobenthic diversity and structure. We used nested sampling designs in the intertidal and subtidal habitats, including horizontal spatial scales ranging from few centimetres to 1000s of metres along the rocky shore of Fildes Peninsula, King George Island. In both intertidal and subtidal habitats, univariate and multivariate analyses showed a marked vertical zonation in taxon richness and community structure. These patterns depended on the horizontal spatial scale of observation, as all analyses showed a significant interaction between height (or depth) and the finer spatial scale analysed. Variance and pseudo-variance components supported our prediction for taxon richness, community structure, and the abundance of dominant species such as the filamentous green alga *Urospora penicilliformis* (intertidal), the herbivore *Nacella concinna* (intertidal), the large kelp-like *Himantothallus grandifolius* (subtidal), and the red crustose red alga *Lithothamnion* spp. (subtidal). We suggest that in coastal ecosystems strongly governed by physical factors, fine-scale processes (e.g. biotic interactions and refugia availability) are still relevant for the structuring and maintenance of the local communities. The spatial patterns found in this study serve as a necessary benchmark to understand the dynamics and adaptation of natural assemblages in response to observed and predicted environmental changes in Antarctica.

## Introduction

Processes that operate at different spatial scales influence the occurrence and abundance of natural species [1], [2]. Thus, identifying the relevant scales of biological variation is an essential prerequisite before proposing predictive models for community dynamics, conservation, and management [3]–[5]. Particularly for highly dynamic systems, such as the Antarctic shores, the analysis of scale-dependent variation can add accuracy to our understanding of how natural assemblages response to natural and anthropogenic impacts (e.g. [6]).

Spatial patterns of species distributions have been traditionally analysed along steep environmental gradients. For example, the large variability in factors such as desiccation and temperature strongly influences the vertical distribution of intertidal organisms [7]. Similarly, attenuation of solar radiation and water movement stimulate the formation of vertical patterns in subtidal communities [8]. However, these patterns in community structure and diversity can vary across horizontal scales of observation, which can confound our conclusions about biological patterns along the most obvious environmental gradients [9], [10]. As an emerging pattern, several studies focused on intertidal and subtidal communities suggest that local processes, including physical disturbance [11], competition [12], [13], availability of refugia [14], and consumption [15], [16] generate a high amount of horizontal biological variability at fine scales of observation (i.e. cm to few metres between sampling units [14], [17]–[19]).

Along Antarctic shores, topographic habitat features can generate fine-scale horizontal variability across vertical environmental stress gradients [20]. Broad seasonal changes in ice cover, salinity, UV radiation, and temperature, impose harsh environmental conditions for intertidal and shallow-subtidal Antarctic assemblages [21]–[24]. Moreover, Antarctic subtidal habitats are characterised by sharp gradients in light availability and seasonal impact of ice scouring between 2 and 10 m depth, which determine the vertical distribution of dominant brown and red algae [20], [23], [25]–[27]. Therefore, milder environmental conditions in structurally complex habitats, such as tide pools and rock interstices, might facilitate the establishment and maintenance of a moderately high biodiversity observed along the vertical stress gradients [28], [29]. Recent observations indicate, in addition, that intertidal and subtidal habitats can support similar numbers of species [30], likely as a function of refugia availability in the former habitat [28].

In addition to substrate characteristics, biotic interactions also influence the fine-scale spatial variability in species abundances and diversity of Antarctic shores [31]. For example, grazing by the limpet *Nacella concinna* shapes the colonisation and abundance of macrobenthic algal species [31]–[34]. The effects of grazing and microhabitats on benthic diversity suggest that fine-scale processes might significantly mediate the effects of the vertical environmental stress gradients on a suite of biological processes (e.g. reproductive output, recruitment, physiological performance, and primary productivity) at intertidal and shallow subtidal zones, shaping the local diversity and community structure. Therefore, assessing the scale-dependent patterns of spatial variability of benthic diversity can help us to understand the functioning and stability of Antarctic coastal ecosystems in predicted climate change scenarios.

Herein, we determined the vertical and horizontal variability in taxon richness and community structure of rocky intertidal and subtidal systems from the Antarctic shores of King George Island, South Shetland Islands. Separately for each habitat (intertidal and subtidal), we tested the general hypothesis that fine-scale processes significantly influence the effects of the vertical environmental stress gradients on coastal diversity and community structure. To test this prediction, we used in each habitat a nested sampling design to quantify species abundances across multiple horizontal spatial scales.

## Material and Methods

**Figure 1:**
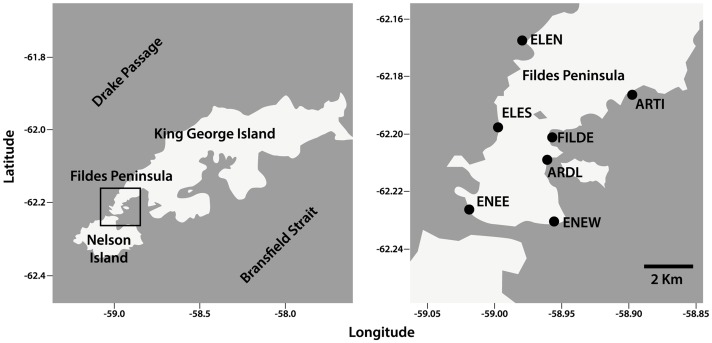
Study shores in Fildes Peninsula. Shore abbreviations are as in Table 1.

The study was conducted as part of the activities carried out by the Algas Antárticas working group at the Universidad Austral de Chile and approved by the Instituto Antártico Chileno (INACH) in accordance with the Protocol on Environmental Protection to the Antarctic Treaty. No additional permit was required because non-destructive samplings were conducted. Moreover, the sampling areas were located outside of the Antarctic Specially Protected Areas. Estimations of species abundances were conducted in Fildes Peninsula, King George Island (Fig. 1), during the austral summer (January) 2013. Glaciers permanently cover more than 90% of the island’s surface. The surface of Fildes Bay (14 km long and 6 – 14 km wide) and the coastal zones regularly freeze in austral winter, from late July to mid-September; after late October, the sea ice starts cracking and floating ices reach the shore [29], [34], [35]. Intertidal sessile assemblages in King George Island are characterised by red algae such as *Iridaea cordata*, brown algae such as *Adenocystis utricularis*, and green algae such as *Urospora penicilliformis;* the intertidal grazer assemblage is dominated by the limpet *Nacella concinna*, followed by the small littorinids *Laevilacunaria antarctica* and *Laevilitorina umbilicata* [27], [34]. The subtidal sessile assemblages are characterised by brown algae such as *Himantothallus grandifolius* and *Desmarestia* spp., red algae such as *Trematocarpus antarcticus* and *Plocamium cartilagineum*, and several species of bryozoans, ascidians, and sponges. The grazer *N. concinna* and the predatory sea star *Odontaster validus* can be frequently found in subtidal habitats [27], [34].

**Figure 2:**
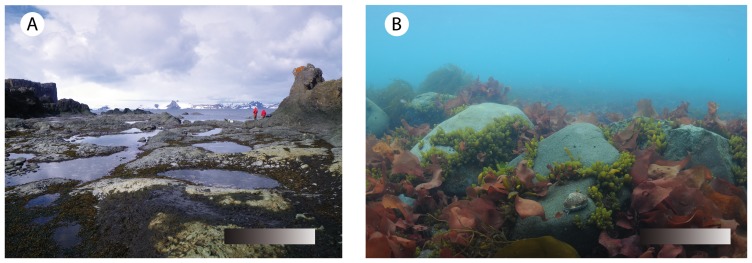
Coastal assemblages in Fildes Peninsula showing spatial patterns of dominant algal species. Scale-dependent spatial patterns of species abundances were determined along intertidal (a) and subtidal (b) rocky shores. Scale bars represent 1 and 0.1 m for intertidal and subtidal habitats, respectively. Photo credits: Nelson Valdivia (a) and Dirk Schories (b).

In the intertidal and subtidal habitats (Fig. 2), we used nested sampling designs in which six and four shores, respectively, were randomly selected along the coast of Fildes Peninsula (Table 1). Shores were of ca. 200 m in alongshore length and separated by >1000 m. In each shore, three sites, separated by few 100 s of metres, were randomly located. Within each site, two patches of substratum separated by 10 s of metres were randomly located. Within each patch, three 50 × 50 cm quadrats were randomly located at the low, mid-, and high intertidal; four quadrats were located at the shallow (5 to 10 m depth) and deep (25 to 30 m depth) subtidal. The sampling plots were restricted to areas of gently slopes, but covered different microhabitats, including emergent rocks, boulders, and shallow tide pools.

**Table 1:** List of sampling shores analysed in this study. Latitude and longitude are expressed as decimal degrees.

	Shore name	Code	Latitude	Longitude	Habitat
1	Elefantes North	ELEN	−62.174	−58.970	Intertidal
2	Elefantes South	ELES	−62.202	−59.001	Intertidal
3	Estrecho Nelson West	ENEW	−62.240	−58.970	Intertidal
4	Estrecho Nelson East	ENEE	−62.232	−58.992	Intertidal, subtidal
5	Fildes	FILDE	−62.200	−58.945	Intertidal, subtidal
6	Artigas	ARTI	−62.188	−58.871	Intertidal, subtidal
7	Ardley Peninsula	ARDL	−62.229	−58.941	Subtidal

The habitat (intertidal, subtidal, or both) where observations were conducted at each shore is provided.

On each intertidal patch of substratum, we used the upper limit of species occurring highest on the shore as appropriate indicator of the upper intertidal boundary, because their upper distribution limits represent a summary of the local wave regime [36]. These taxa were the green alga *Ulothrix* sp. and the red alga *Porphyra endiviifolium*. Once the upper boundary was determined on each shore, we divided the intertidal range in three zones of equal vertical extent (high, mid-, and low zones).

### Sampling procedure

All seaweeds and invertebrates (> 1 mm) occurring on each intertidal and subtidal quadrat were identified in situ. Because of small size or morphological overlap with similar species, a few taxa were classified to the lowest possible taxonomic level (usually genus), as normally done in field studies that identify all producers and consumers simultaneously [37], [38].

For each quadrat, we measured the percentage cover of each sessile species using a 50 × 50 cm frame divided in 25 equal fields with monofilament line. Cover values were obtained from the projection of three-dimensional structures to the plane of the sampling frame. Mobile species were quantified as number of organisms per m^2^. We used the same sampling procedure across intertidal and subtidal habitats. These data are publicly available as Supporting Information files, Data S1 and S2 (intertidal and subtidal habitats, respectively)—explanatory information for the intertidal and subtidal datasets is provided in Data S3 and S4, respectively.

Taxon richness was calculated as the total number of taxa identified on each quadrat. Community structure was represented as pairwise Bray-Curtis dissimilarities. In order to include both, percentage covers and densities (i.e. sessile and mobile taxa, respectively) in the analyses of community structure, data of each taxon were first transformed to proportions of the maximum observed for each taxon across the shores. In this way, all cover and density data ranged between 0 (absence of species in a given quadrat) and 1 (maximum cover or density in the study area).

### Statistical analyses

Separately for the intertidal and subtidal habitats, the hypothesis that fine-scale processes significantly influence the effects of the vertical environmental stress gradients on taxon richness and community structure was tested with univariate and multivariate mixed-model analyses of variance (ANOVA and PERMANOVA, respectively). We conducted ANOVA on species richness and PERMANOVA on the Bray-Curtis dissimilarities calculated from proportion-transformed abundance data [39]. The mixed models included shore, site (nested within shore), and patch (nested within site) as random factors, and height or depth as fixed factor. In these analyses, a significant interactive effect of height (or depth) by the patch scale on taxon richness (or community structure) would support our prediction. Homogeneity of variance of taxon richness was graphically explored by means of residuals-vs.-fits and normal Q-Q plots. Patterns of variation in community structure were visualised in multi-dimensional scaling (MDS) ordinations, based on the Bray-Curtis dissimilarities calculated from the proportion-transformed abundance data.

We calculated the contribution of each taxon to the spatial variation in community structure with Similarity Percentage (SIMPER) routines. In this procedure, we calculated Bray-Curtis dissimilarities among replicate plots, between groups, and within groups in the entire dataset of proportion-transformed abundance data. The average between-group dissimilarities were then broken down into separate contributions from each taxon [40]. Taxon contributions were calculated separately for height or depth.

In order to determine patterns of horizontal variation in taxon richness, community structure, and the abundance of representative species detected with SIMPER routines, we estimated variance and pseudo-variance components (from ANOVA and PERMANOVA, respectively) for each intertidal height and subtidal depth. For each random factor, variance and pseudo-variance components were estimated as the difference between its mean square (MS) and the MS of the term immediately below in the nested hierarchy. In case of negative variance components, these were set to zero. PERMANOVA were conducted in PRIMER v6 with the PERMANOVA+ add-on [41], and all the other analyses were conducted in R version 3.02 [42].

## Results

**Figure 3:**
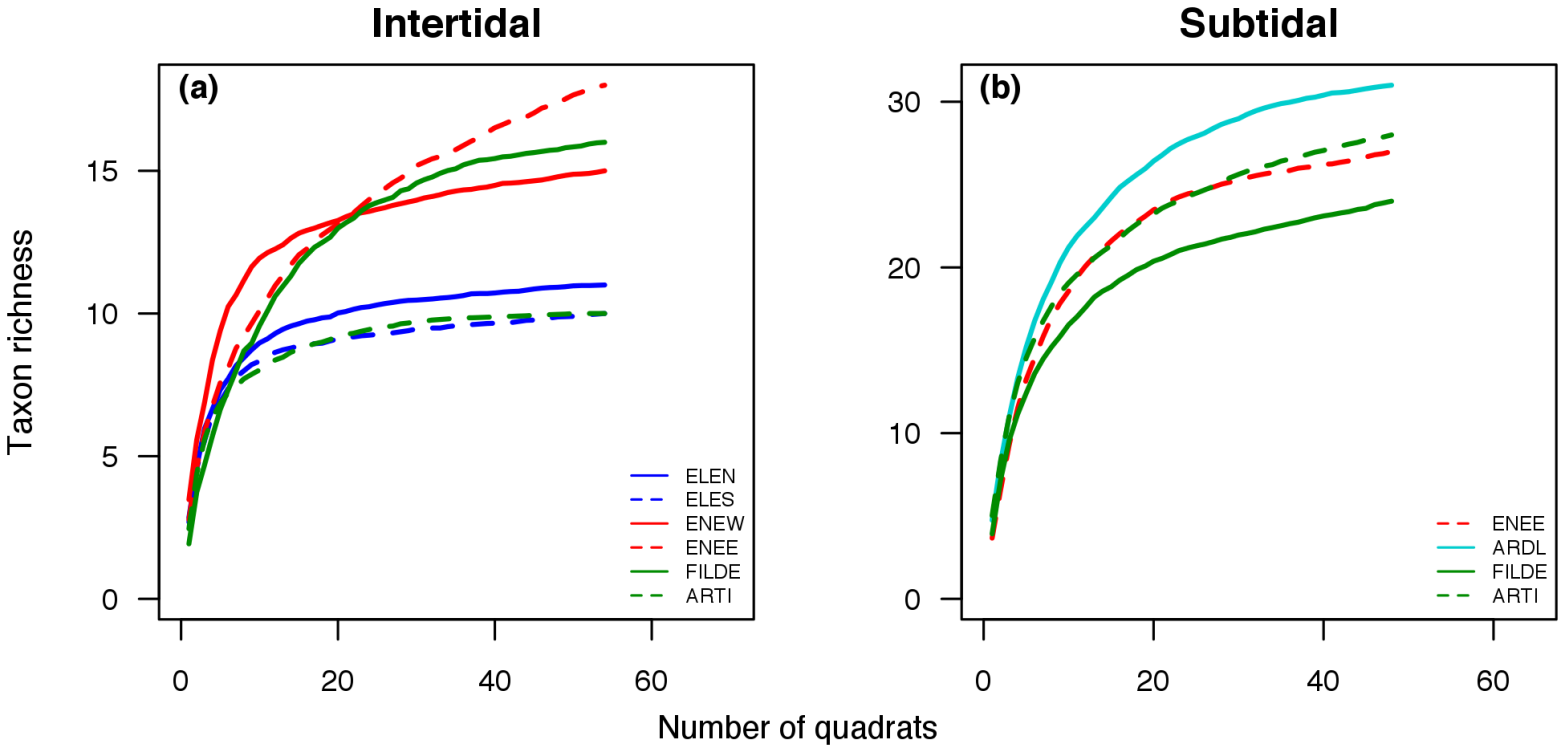
Species-accumulation curves for each intertidal (a) and subtidal (b) study shore. See Table 1 for shore locations and abbreviations. Note different scales of Y-axes.

Across the study area, we observed 24 and 40 taxa in the intertidal and subtidal habitats, respectively. Fourteen macroalgal taxa were found in the intertidal and 25 in the subtidal habitat. Invertebrates occurred with 10 and 15 taxa in the intertidal and subtidal habitats, respectively. For the intertidal habitats, the shores with the highest richness were those facing Nelson Island (i.e. ENEW and ENEE, Fig. 3a). The lowest richness was observed on the wave-exposed shores facing the Drake Passage (i.e. ELEN and ELES, Fig. 3a). The wave-sheltered shores showed contrasting patterns, with low (ARTI) and high (FILDE) richness values (Fig. 3a). The subtidal habitats displayed more homogeneous richness patterns at the shore-scale, with ARLD and FILDE harbouring the highest and lowest number of taxa, respectively (Fig. 3b).

**Figure 4:**
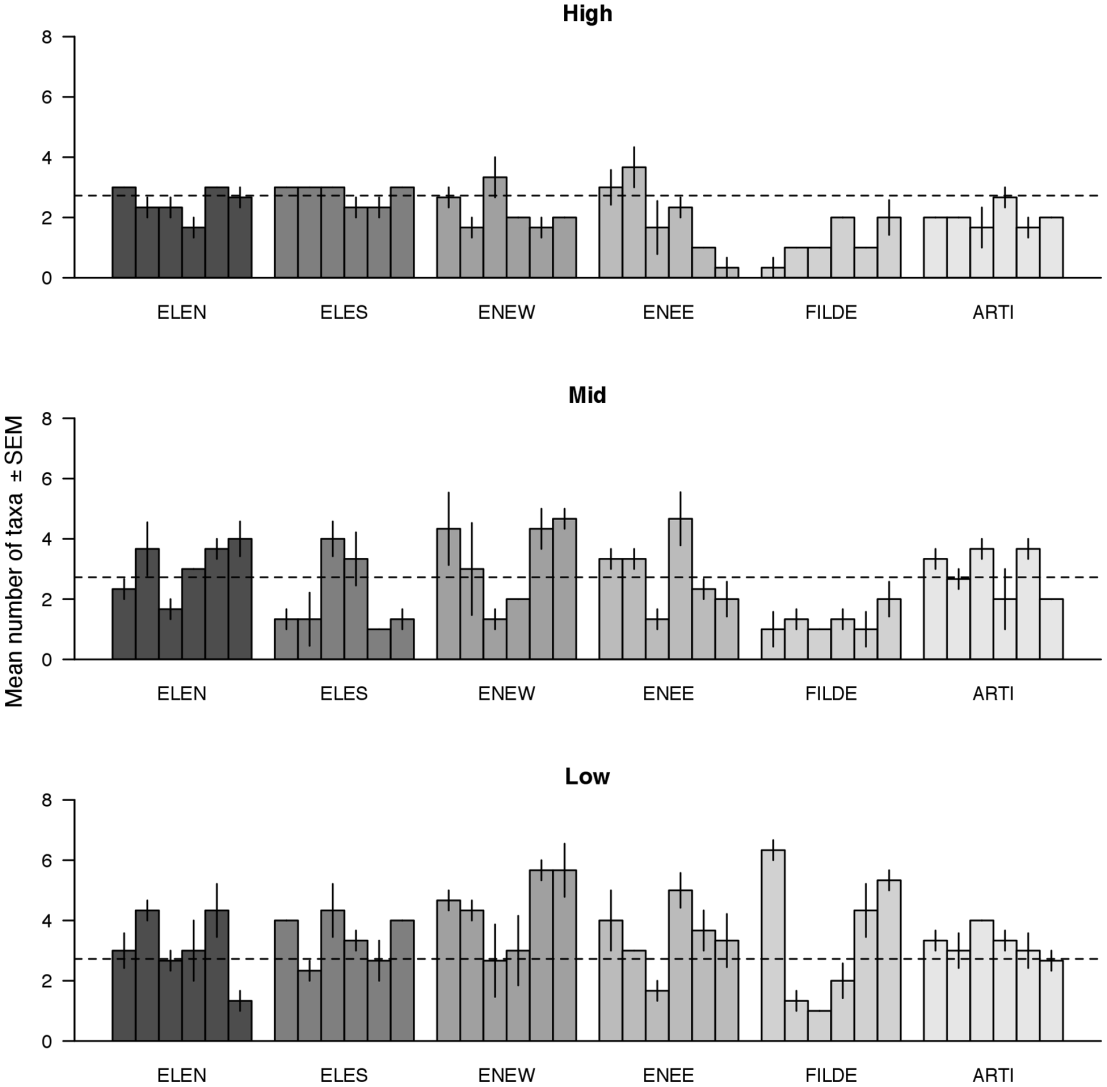
Local-scale patterns of taxon richness for each intertidal shore and elevation (high, mid-, and low intertidal) in Fildes Peninsula. Each bar represents a ca. 3Table 1 for shore location and abbreviations.

**Figure 5:**
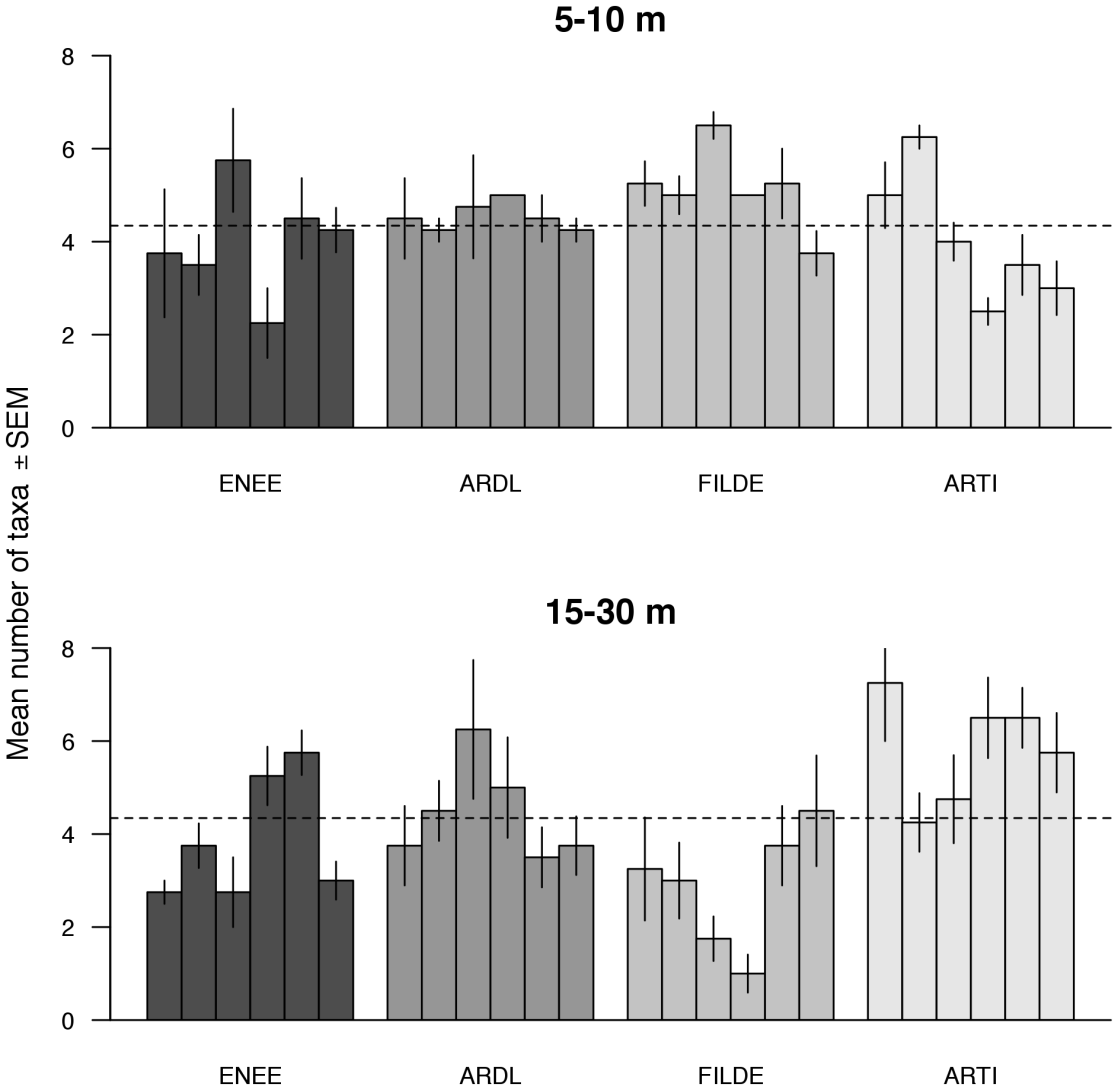
Local-scale patterns of taxon richness for each subtidal shore in Fildes Peninsula. Each bar represents a ca. 3Table 1 for shore location and abbreviations.

Local patterns of intertidal taxon richness showed high levels of vertical and horizontal variability (Fig. 4). Taxon richness tended to decrease with the vertical environmental stress gradient (i.e. from low to high intertidal heights). These patterns, however, varied between scales of observations, as habitat patches showed wide differences in taxon richness (Fig. 4). Accordingly, ANOVA indicated a significant interactive effect of height by patch scale on taxon richness (Table S1). For the subtidal habitat, the vertical zonation in taxon richness was less clear than for the intertidal habitat (Fig. 5). Horizontal variation, nevertheless, was also important in the subtidal. Taxon richness tended to increase from shallow to deep waters at ARDL and ARTI, but it showed an inverse trend at FILDE; at ENEE, taxon richness did not show a well-defined vertical pattern of variation (Fig. 5). The ANOVA results supported these patterns, as a significant interaction between depth and patch scale was detected (Table S1).

**Figure 6:**
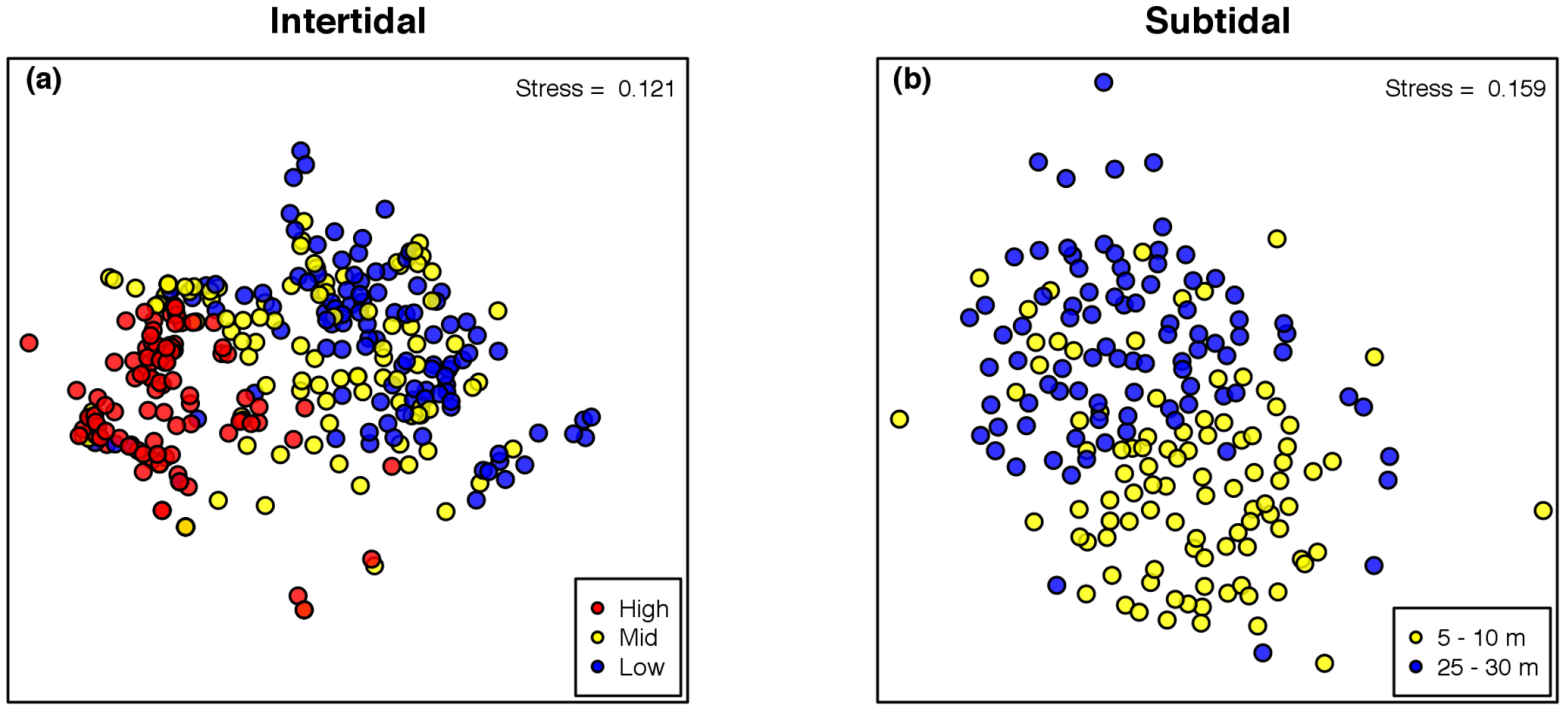
Multi-dimensional scaling ordinations (MDS) for the intertidal (a) and subtidal (b) habitats. For the intertidal and subtidal systems, the ordinations distinguish between intertidal elevations (high, mid, and low) and subtidal depths (5–10 m and 25–30 m), respectively. Symbols represent single quadrats.

The MDS ordination discriminated the assemblages according the dissimilarities among the intertidal heights (Fig. 6a) and between the subtidal depths (Fig. 6b). For the intertidal habitat, the mid-zone appeared in between the low and high zones (Fig. 6a). Nevertheless, we observed a high overlap between the mid- and low intertidal heights and between the shallow and deep subtidal levels (Fig. 6). The PERMANOVA results supported the influence of the horizontal variability on the effects of the vertical stress gradients on community structure, as the interactive effect of both factors was significant (Table S1).

**Figure 7:**
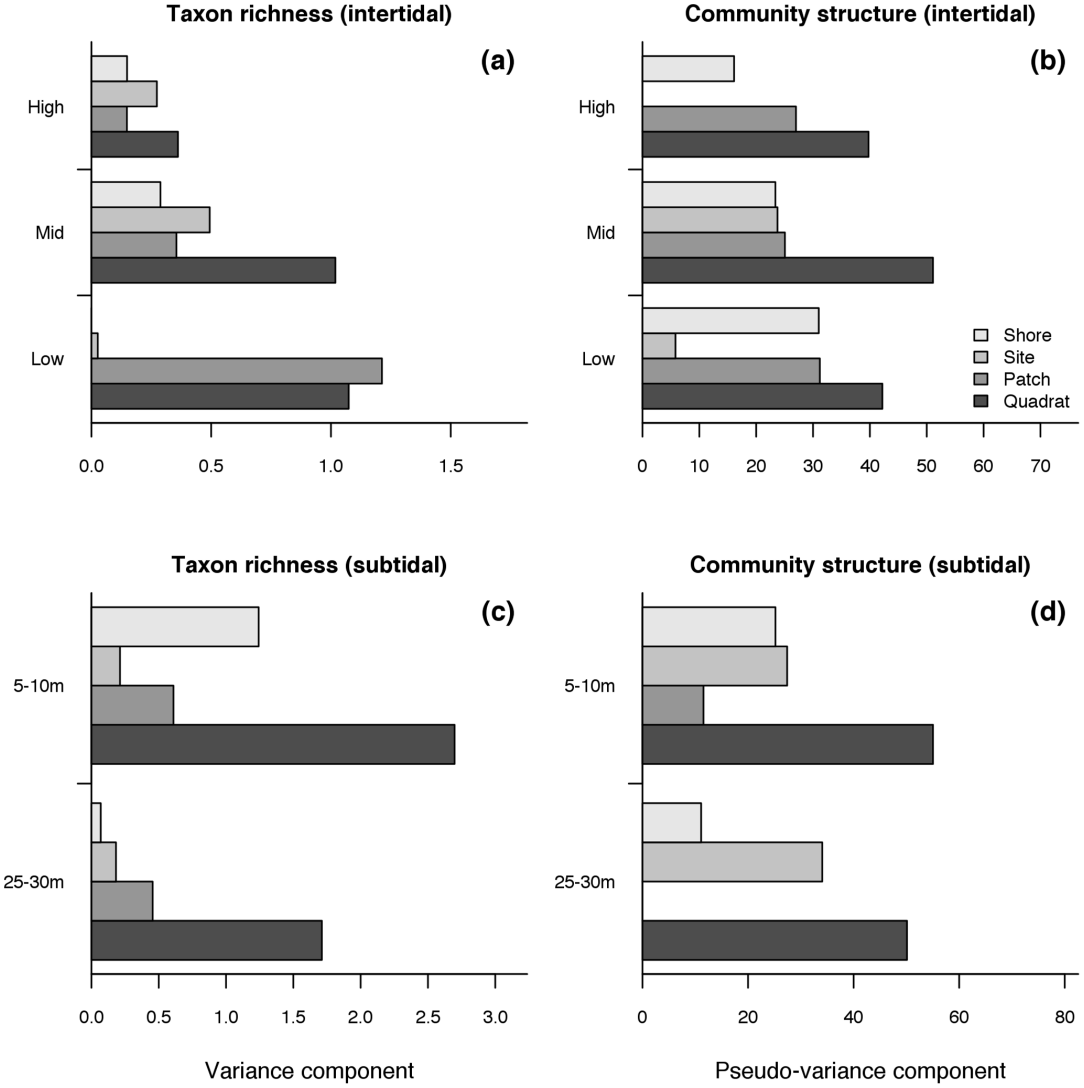
Variance components of taxon richness and pseudo-variance components of Bray-Curtis (%) dissimilarities across scales of spatial variability and habitat (intertidal and subtidal). Pseudo-variance components were square-root transformed to present actual Bray-Curtis values.

Variance components of taxon richness decreased from low to high intertidal heights (Fig. 7a). In addition, the higher variance components were those observed at the finer scale or variation (i.e. the quadrat scale of tens of cm to few metres, Fig. 7a). The community structure showed a similar pattern of variation, with pseudo-variance components peaking at the mid-intertidal height and at the quadrat scale (Fig. 7b). In the subtidal habitat, variance components of taxon richness decreased from shallow to deep waters and were highest at the quadrat scale of observation (Fig. 7c). Subtidal pseudo-variance components also decreased from shallow to deep waters and peaked at the quadrat scale (Fig. 7d).

According to the SIMPER analyses, eight taxa explained up to the 90% of the dissimilarity between the high and mid-intertidal, and twelve taxa were the most important in discriminating the mid- and low intertidal zones (Table 2). The contrasts suggested that the high intertidal areas were characterised by the filamentous green algae *Urospora penicilliformis* and *Ulothrix* sp. and the red alga *Porphyra endiviifolium*; the mid-intertidal showed high abundances of *U. penicilliformis,* the brown alga *Adenocystis utricularis,* the red algae *Palmaria decipiens* and *Iridaea cordata*, *Ulothrix* sp., and the crustose red algae *Lithothamnion* spp., in addition to *Nacella concinna* and littorinid snails; the low intertidal was characterised by *P. decipiens, I. cordata, A. utricularis,* the green alga *Monostroma hariotii,* and *N. concinna* (Table 2).

**Table 2:** Similarity Percentage (SIMPER) analyses for intertidal taxa: mean abundances (proportion-transformed data) of intertidal taxa in each intertidal height.

Taxon	Mean abundance		Cumulative contribution
	**High**	**Mid**	
*Urospora penicilliformis*	0.27	0.20	32.5
*Ulothrix* sp.	0.19	0.05	55.2
*Porphyra endiviifolium*	0.09	0.00	64.4
*Adenocystis utricularis*	0.00	0.07	73.1
*Palmaria decipiens*	0.00	0.05	78.5
*Iridaea cordata*	0.00	0.04	82.7
*Lithothamnion* spp.	0.00	0.04	86.8
Laevilitorininae spp.	0.00	0.03	90.6
	**Mid**	**Low**	
*Urospora penicilliformis*	0.20	0.07	19.2
*Adenocystis utricularis*	0.07	0.07	32.1
*Palmaria decipiens*	0.05	0.10	44.6
*Iridaea cordata*	0.04	0.10	55.6
*Nacella concinna*	0.03	0.07	63.8
*Ulothrix* sp.	0.05	0.03	71.2
*Lithothamnion* spp.	0.04	0.03	77.4
*Monostroma hariotii*	0.01	0.03	81.4
Laevilitorininae spp.	0.03	0.00	84.2
*Acrosiphonia arcta*	0.00	0.03	86.4
*Gainia mollis*	0.01	0.02	88.4
*Clathromorphum* sp.	0.01	0.02	90.2

Cumulative contribution (%) of each taxon to the between-group dissimilarities is provided (90% cut-off).

In the subtidal habitat, 25 taxa explained up to 90% of the between-depth dissimilarity (Table 3). The shallow waters showed high abundances of *Lithothamnion* spp., the brown alga *Desmarestia menziesii, P. decipiens, N. concinna,* and the sea star *Odontaster validus;* the deeper layer was characterised by the brown alga *Himantothallus grandifolius,* the red algae *Plocamium cartilagineum, Trematocarpus antarcticus,* and *Picconiella plumosa,* and an unidentified Ectoprocta species (Table 3).

**Table 3:** SIMPER analyses for subtidal taxa: mean abundances (proportion-transformed data) of subtidal taxa in each depth.

Taxon	Mean abundance		Cumulative contribution
	5–10 m	25–30 m	
*Lithothamnion* spp.	0.19	0.07	9.5
*Himantothallus grandifolius*	0.09	0.11	16.9
*Plocamium cartilagineum*	0.09	0.10	23.1
*Odontaster validus*	0.10	0.06	28.5
*Desmarestia menziesii*	0.07	0.05	33.9
*Trematocarpus antarcticus*	0.04	0.09	38.7
Unidentified Ectoprocta sp. 2	0.02	0.09	43.5
*Palmaria decipiens*	0.09	0.02	47.8
*Nacella concinna*	0.08	0.02	51.8
*Desmarestia anceps*	0.06	0.03	55.8
Unidentified Asteroidea sp. 1	0.03	0.06	59.5
*Pantoneura plocamioides*	0.06	0.04	62.9
*Sterechinus neumayeri*	0.02	0.04	66.3
*Gigartina skottsbergii*	0.04	0.03	69.5
*Picconiella plumosa*	0.02	0.06	72.5
*Ophionotus victoria*	0.01	0.05	75.0
*Ascoseira mirabilis*	0.05	0.01	77.4
Unidentified Ascidiacea sp. 1	0.01	0.06	79.9
Unidentified Demospongiae sp. 1	0.04	0.02	81.9
*Curdiea racovitzae*	0.02	0.02	83.7
*Phaeurus antarcticus*	0.02	0.02	85.2
Unidentified Asteroidea sp. 2	0.03	0.01	86.7
Unidentified Rhodophyta sp. 1	0.00	0.04	88.0
*Desmarestia antarctica*	0.01	0.02	89.4
*Monostroma hariotii*	0.02	0.00	90.5

Cumulative contribution (%) of each taxon to the between-group dissimilarities is provided (90% cut-off).

**Figure 8:**
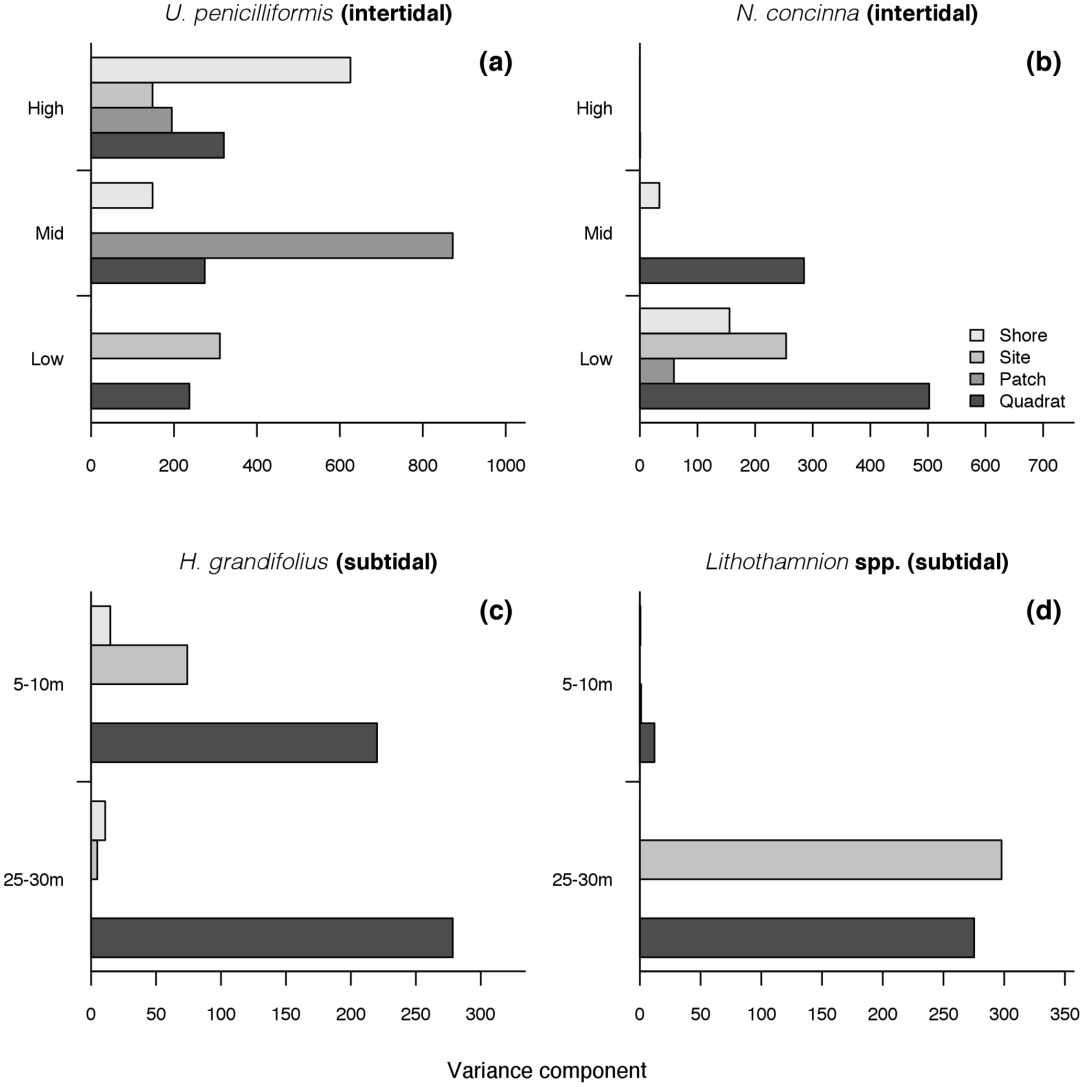
Variance components of dominant taxa across scales of spatial variability and habitats (intertidal and subtidal).

Finally, the variance components of representative intertidal taxa in the SIMPER analyses showed in general the highest variances at the finer scales of observation (Fig. 8). *Urospora penicilliformis* was highly variable at the scale of shores (high intertidal, Fig. 8a), but the highest variance component was observed at the patch-scale (mid-intertidal). Excepting for *U. penicilliformis*, the representative species were highly variable at the quadrat-scale. *Nacella concinna* showed the highest variance components in the mid- and low intertidal heights (Fig. 8b), and *H. grandifolius* and *Lithothamnion* spp. displayed a strong variability at deeper waters (Figs. 8c and 8d). *Lithothamnion* spp. was highly variable at the scale of site (Fig. 8d).

## Discussion

The results showed significant vertical variations in diversity, community structure, and the abundance of dominant species in Fildes Peninsula, Antarctica. For the intertidal and subtidal assemblages, there were species characterising the different levels of the vertical environmental stress gradients. These patterns were scale-dependent, and variance and pseudo-variance components supported the prediction that fine-scale processes are relevant for the diversity, abundance, and structure of these assemblages. Our results agree with previous studies pointing to the pervasive nature of fine-scale variability in spatial patterns of species abundances and diversity showed elsewhere [18], [19], [43]. Local processes involving consumption and topographic features may explain the high horizontal variability observed at fine spatial scales.

Our results agreed with the general pattern of species zonation in intertidal and subtidal habitats [7], [43]. In the intertidal habitat, desiccation risk, temperature, and UV radiation exposure significantly increase from low to high intertidal heights and define the upper vertical limits of species with differing environmental tolerances [27], [44]–[46]. In the subtidal habitat, diverse water column processes such as light attenuation, water movement, sedimentation, and changes in salinity trigger vertical patters of community composition [8], [47], [48]. In the case of algae, light availability for photosynthesis strongly defines the vertical distribution in the subtidal areas; at shallow-subtidal and intertidal bottoms, the impact of high solar radiation (including UV radiation) can become relevant on Antarctic shores [27]. Therefore, and despite the extreme environmental conditions described for Antarctic coasts, marine species at Fildes Peninsula and elsewhere (see ref. [28]) are able to colonise the intertidal and shallow-subtidal habitats and generate significant zonation patterns.

The steep environmental gradients in intertidal and shallow-subtidal environments determine changes in the strength of biotic interactions [49]. For example, the strength of negative interactions is predicted to decrease with increasing environmental stress [49], [50]. Moreover, predation effects on intertidal species have been shown to significantly vary along gradients of thermal stress [51]. Considering the comparatively harsh environmental conditions of Antarctic shores, it is likely that biotic interactions among marine organisms could have been strongly limited to the low intertidal and the subtidal habitats. In our study, the highest abundance of the limpet *Nacella concinna* was observed at the low intertidal and shallow subtidal habitats. Grazing is a structuring force in Antarctic intertidal and shallow subtidal communities [34], [52]. In addition, *N. concinna* can mediate the effects of broad-scale stressors, such as UV radiation, on macrobenthic diversity [32]. Accordingly, grazing by *N. concinna* could well have influenced the lower distribution limit of intertidal algae, in agreement with early models of intertidal zonation [44], [53].

The vertical zonation patterns discussed above depended on the spatial scale of observation, which is well in line with studies conducted in temperate [18], [19] and sub-Antarctic shores [54], [55]. In particular, the effects of intertidal height and subtidal depth on diversity and community structure significantly varied among the smallest sampling units in our study. Processes involving competition, consumption, and local habitat characteristics have been proposed as explanations for fine-scale patterns of variability [15]. As expressed in the previous paragraph, grazing activity of intertidal and subtidal limpet populations control the re-colonisation and structure of benthic sessile assemblages in Antarctica [32]–[34]. According to the stressful conditions of the Antarctic habitats, the availability of refugia (e.g. tide pools, boulders) could favour the development of local populations and thus biotic interactions. Habitat complexity can ameliorate the environment and provide more suitable conditions for the establishment and maintenance of diversity (e.g. [56]). For instance, habitat complexity in the Antarctic intertidal affects the diversity and structure of benthic assemblages, with diversity increasing towards more complex habitats [28]. Therefore, fine-scale processes, such as biotic interactions (e.g. grazing) and habitat complexity (e.g. substrate heterogeneity), could well interact and superimpose a patchy distribution of species on most obvious environmental stress gradients.

Our results were consistent between the intertidal and subtidal habitats. In addition, the high amount of fine-scale variability observed in our study is well supported by previous records (reviewed in [17]), suggesting that fine-scale variation is a common and central characteristic of coastal assemblages. Biotic interactions have been proposed as general processes generating fine-scale variability across biogeographic regions and habitats. In our study, predation by sea stars could also have contributed to the fine-scale variability observed in the shallow subtidal; for example, the predatory sea star *Odontaster validus* scored a high rank in the SIMPER analyses. According to this suggestion, it is already established that variation in consumption (grazing and predation) could either stimulate or dampen the spatial heterogeneity in the distribution of resource species [16], [57]. Manipulative experiments suggest, in addition, that grazers and predators have determinist effects on the local spatial variability and functioning of ecosystems over broad, geographic, scales [58]–[60]. Albeit the direction of effects may vary according to local conditions and species pools, consumption appears as a pivotal process for the structure and stability of natural ecosystems.

Our study should be considered in context with broader spatiotemporal scales of observations. First, the study was conducted in a limited area of the South Shetland Archipelago. So, it is likely that the effects of broad-scale processes, like glacier influence [61], could not have been fully captured in our sampling design. Second, and since sea ice melting and the intertidal assemblage recovery in Fildes Peninsula starts in late October [35], our sampling (conducted in January) could have included assemblages in an intermediate successional stage. Nevertheless, experiments using field-grown macroalgal assemblages and deployed during different periods in the Antarctic summer show virtually the same general pattern [32].

Overall, our analyses allowed us to detect consistent patterns of biological variability across intertidal and subtidal habitats, which are usually studied separately. We propose that grazing and refugia availability are likely relevant factors at the fine-scale along the coast of Fildes Peninsula. These processes have been shown to significantly influence the structure and stability of natural assemblages across several marine and terrestrial ecosystems, and their effects can scale to broader spatial scales (e.g. [60]). Local processes, therefore, should now be considered in models of community- and ecosystem-level responses to large-scale phenomena, such as natural and anthropogenic impacts. The patterns here documented can improve our predictions of how the dynamic assemblages in Antarctica will respond to the environmental changes predicted for this valuable ecosystem.

## Supporting Information

Table S1 **Results of analyses of variance on scale-dependent spatial patterns of taxon richness (ANOVA) and community composition (PERMANOVA) in Península Fildes.**
(DOCX)

Data S1 **Entire dataset of percentage covers and densities of intertidal macrobenthic species from Fildes Peninsula, King George Island.**
(CSV)

Data S2 **Dataset of percentage covers and densities of subtidal macrobenthic species from Fildes Peninsula, King George Island.**
(CSV)

Data S3 **Metadata explaining each column in Data S1.**
(CSV)

Data S4 **Metadata explaining each column in Data S2.**
(CSV)
