# Supplementary material for: Up, Down, and All Around: Scale-Dependent Spatial Variation in Rocky-Shore Communities of Fildes Peninsula, King George Island, Antarctica
Source: PLoS One. 2014 Jun 23;9(6):e100714. doi: 10.1371/journal.pone.0100714 (PMC4067381; doi:10.1371/journal.pone.0100714)
Supplement: Table S1 — Results of analyses of variance on scale-dependent spatial patterns of taxon richness (ANOVA) and community composition (PERMANOVA) in Península Fildes. (DOCX) [file pone.0100714.s001.docx]

**Table S1** Results of analyses of variance on scale-dependent spatial patterns of taxon richness (ANOVA) and community composition (PERMANOVA) in Península Fildes. PERMANOVA were computed on Bray-Curtis dissimilarities calculated from proportion-transformed abundance data. **^*^**p < 0.05, **^**^**p < 0.01, **^***^**p < 0.001.

| Habitat | Source of |  | Taxon richness | | | Community structure | | |
| --- | --- | --- | --- | --- | --- | --- | --- | --- |
|  | variation | df | MS | F |  | MS | Pseudo-F |  |
| Intertidal | Height = H | 2 | 52.8 | 11.9 | **^***^** | 10^9^ | 11.3 | **^***^** |
|  | Shore = Sh | 5 | 9.9 | 1.7 |  | 28891 | 3.9 | **^***^** |
|  | Site(Sh) = Si(Sh) | 12 | 5.9 | 1.7 |  | 7338 | 12.3 |  |
|  | H x Sh | 10 | 4.4 | 1.3 |  | 9584 | 21.5 | **^***^** |
|  | Patch(Si(Sh)) = Pa(Si(Sh)) | 18 | 3.5 | 4.3 | **^***^** | 5960 | 29.9 | **^***^** |
|  | H x Si(Sh) | 24 | 3.5 | 1.7 |  | 4454 | 12.7 | **^*^** |
|  | H x Pa(Si(Sh)) | 36 | 2.0 | 2.5 | **^***^** | 3502 | 17.6 | **^***^** |
|  | Residual | 216 | 0.8 |  |  | 1991 |  |  |
|  |  |  |  |  |  |  |  |  |
| Subtidal | Depth = D | 1 | 1.3 | 0.04 |  | 72525 | 40.3 | **^*^** |
|  | Shore = Sh | 3 | 10.6 | 2.11 |  | 21444 | 20.4 | **^*^** |
|  | Site(Sh) = Si(Sh) | 8 | 5.0 | 2.68 |  | 10508 | 38.6 | **^***^** |
|  | D x Sh | 3 | 32.7 | 4.80 | **^*^** | 17990 | 16.8 |  |
|  | Patch(Si(Sh)) = Pa(Si(Sh)) | 12 | 1.9 | 0.85 |  | 2724 | 1.0 |  |
|  | D x Si(Sh) | 8 | 6.8 | 1.00 | **^*^** | 10731 | 3.3 | **^***^** |
|  | D x Pa(Si(Sh)) | 12 | 6.8 | 3.09 | **^***^** | 3222 | 11.6 | **^*^** |
|  | Residual | 144 | 2.2 |  |  | 2770 |  |  |
